# Auditory and Visual Sequence Learning in Humans and Monkeys using an Artificial Grammar Learning Paradigm

**DOI:** 10.1016/j.neuroscience.2017.06.059

**Published:** 2018-10-01

**Authors:** Alice E. Milne, Christopher I. Petkov, Benjamin Wilson

**Affiliations:** Institute of Neuroscience, Henry Wellcome Building, Newcastle University, Framlington Place, Newcastle upon Tyne NE2 4HH, United Kingdom; Centre for Behaviour and Evolution, Henry Wellcome Building, Newcastle University, Framlington Place, Newcastle upon Tyne NE2 4HH, United Kingdom

**Keywords:** structured sequence learning, auditory, visual, macaque, human, comparative

## Abstract

Language flexibly supports the human ability to communicate using different sensory modalities, such as writing and reading in the visual modality and speaking and listening in the auditory domain. Although it has been argued that nonhuman primate communication abilities are inherently multisensory, direct behavioural comparisons between human and nonhuman primates are scant. Artificial grammar learning (AGL) tasks and statistical learning experiments can be used to emulate ordering relationships between words in a sentence. However, previous comparative work using such paradigms has primarily investigated sequence learning within a single sensory modality. We used an AGL paradigm to evaluate how humans and macaque monkeys learn and respond to identically structured sequences of either auditory or visual stimuli. In the auditory and visual experiments, we found that both species were sensitive to the ordering relationships between elements in the sequences. Moreover, the humans and monkeys produced largely similar response patterns to the visual and auditory sequences, indicating that the sequences are processed in comparable ways across the sensory modalities. These results provide evidence that human sequence processing abilities stem from an evolutionarily conserved capacity that appears to operate comparably across the sensory modalities in both human and nonhuman primates. The findings set the stage for future neurobiological studies to investigate the multisensory nature of these sequencing operations in nonhuman primates and how they compare to related processes in humans.

## Highlights

• The evolutionary roots of human language as a multisensory system remain unclear.
• Monkeys and humans were tested using auditory and visual rule-based sequences.
• Comparable patterns of behavioural responses were seen across modalities.
• Monkey and human behavior was largely similar, with subtle differences.
• Primates appear to possess comparable modality-general sequencing mechanisms.

## Graphical abstract

**Figure:**
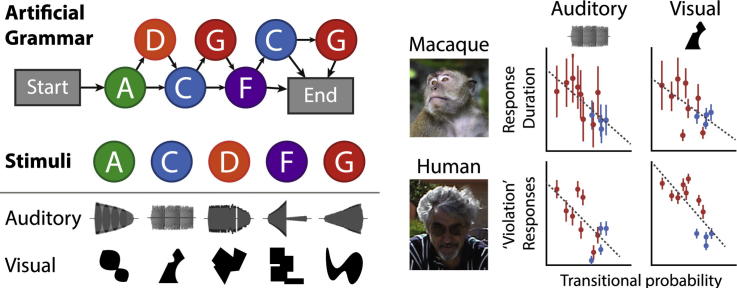

## Introduction

Language transcends the sensory modalities that provide humans with a medium for communication. The same linguistic information can be conveyed by auditory (spoken), visual (written), or somatosensory (Braille) inputs (Liberman et al., 1967, Kavanagh and Mattingly, 1972, De Gelder and Morais, 1995, Bemis and Pylkkänen, 2012). Moreover, when sentences are delivered as spoken words or written text, some of the same brain areas tend to be engaged (for a review see: Price, 2012). Therefore, many of the cognitive processes that support language are thought to be sensory input invariant.

Evidence for evolutionary precursors to human language can be assessed by studying the extent to which nonhuman animal abilities are similarly multimodal (Hauser et al., 2002, Fitch, 2010). There is already evidence to support the notion that the communicative abilities of nonhuman animals are inherently multisensory (Jordan et al., 2005, Budinger et al., 2006, Ghazanfar and Schroeder, 2006, Chandrasekaran et al., 2009, Leavens et al., 2010, Cohen et al., 2011, Romanski, 2012, Soto-Faraco et al., 2012, Ghazanfar and Takahashi, 2014). Moreover, sequence processing abilities have been suggested to tap into an evolutionary precursor to human syntax (Hauser et al., 2002, Saffran et al., 2008, Bickerton and Szathmáry, 2009, Hurford, 2011, Petkov and Wilson, 2012). However, how multisensory the structured sequence processing abilities of nonhuman animals are remains unknown.

Artificial Grammar Learning (AGL) paradigms and statistical learning experiments allow us to study how human and nonhuman animals process the ordering relationships between elements in a sequence. Humans readily learn different types of Artificial Grammars (AGs), irrespective of whether sequences are comprised of visual, auditory or tactile stimuli (Reber, 1967, Fiser and Aslin, 2001, Conway and Christiansen, 2005, Conway and Christiansen, 2006, Saffran et al., 2008). This literature suggests that human sequence processing depends on common operations regardless of the sensory modality in which the sequences are presented. Some studies find evidence that a certain level of sensory-domain specificity is retained in sequencing operations, because humans do not naturally transfer their implicit knowledge about rule-based sequence ordering relationships across sensory modalities (Conway and Christiansen, 2006, Frost et al., 2015, Walk and Conway, 2016). Yet, even if cross-sensory transfer of sequencing knowledge is limited, the pattern of behavioural responses given to auditory and visual sequences can be remarkably similar (Seitz et al., 2007). Altogether, these observations suggest that in humans similar mechanisms operate on the sequences. Possibly there are separate or duplicate processes occurring in parallel across the sensory streams with constraints imposed on cross-modal transfer of sequence ordering knowledge (Conway and Christiansen, 2006, Frost et al., 2015, Walk and Conway, 2016).

Very little is known about how nonhuman animals’ sequence processing abilities compare across sensory modalities and across species. Many species of birds can recognize sequence ordering relationships in the auditory (e.g., Gentner et al., 2006, Herbranson and Shimp, 2008, van Heijningen et al., 2009, Stobbe et al., 2012, Spierings and ten Cate, 2016); and visual modality (e.g., Herbranson and Shimp, 2008, Stobbe et al., 2012) for a review see (ten Cate and Okanoya, 2012). Rats are also able to process structured sequences consisting of visual (Stobbe et al., 2012), auditory (Toro and Trobalón, 2005) or both types of stimuli (Murphy et al., 2008). Several nonhuman primate species are able to recognise certain ordering relationships between stimuli, as reported in a variety of sequence learning experiments in the auditory (tamarins: Fitch and Hauser, 2004, Newport et al., 2004, Saffran et al., 2008; macaques: Hauser and Glynn, 2009, Wilson et al., 2013, Wilson et al., 2015b, marmosets: Wilson et al., 2013; squirrel monkeys: Ravignani et al., 2013) or visual modalities (chimpanzees: Sonnweber et al., 2015). A recent study with chimpanzees identified cross-sensory effects on visual sequence processing (Ravignani and Sonnweber, 2017). The apes were initially trained to select a symmetrical rather than an asymmetrical sequence of visual stimuli (i.e., XYX vs XYY). Subsequently, priming the animals with a previously unheard symmetrical auditory sequence (a sequence of high–low–high tones) produced faster reactions when identifying the symmetrical ‘XYX’ visual stimuli. Priming with asymmetrical sequences (high–low–low tones) had no effect. This study shows that chimpanzees can be influenced by concordant cross-modal information during certain types of sequence processing.

An outstanding question is how human and nonhuman primates learn identically structured sequences consisting of different forms of sensory input and how behavioural responses compare across sensory modalities and species. It is possible that patterns of responses differ not only across the species but also across sensory modalities. In this case, the evidence would point to relatively recent unification of these systems in humans, in ways that differ from other extant primates. Alternatively, if the mechanisms used for sequence processing in a nonhuman primate are similar regardless of the sensory domain, as they are in humans, this would support the notion that evolutionarily conserved sequencing capabilities operate comparably across the sensory modalities and originate prior to the last common ancestor of humans and macaques.

In this study we used an AGL paradigm previously used to study human infants and monkeys in the auditory modality (Saffran et al., 2008, Wilson et al., 2013, Wilson et al., 2015b) to generate sequences of either auditory or visual stimuli. This approach allowed us to assess the pattern of behavioural responses of macaque monkeys and human participants to identical rule-based sequences in the two sensory modalities. The results show that both macaques and humans are sensitive to the adjacent sequence ordering relationships in both visual and auditory sequences. The patterns of responses observed demonstrate considerable correspondences across modalities in humans and monkeys, with overall similarity between the species amidst some more quantitative differences. Altogether, the findings provide evidence for the presence of an evolutionarily conserved system for sequence processing, which appears to similarly processes input from the different sensory modalities in both humans and monkeys.

## Experimental procedures

### Stimuli

#### Artificial grammar

**Fig. 1:**
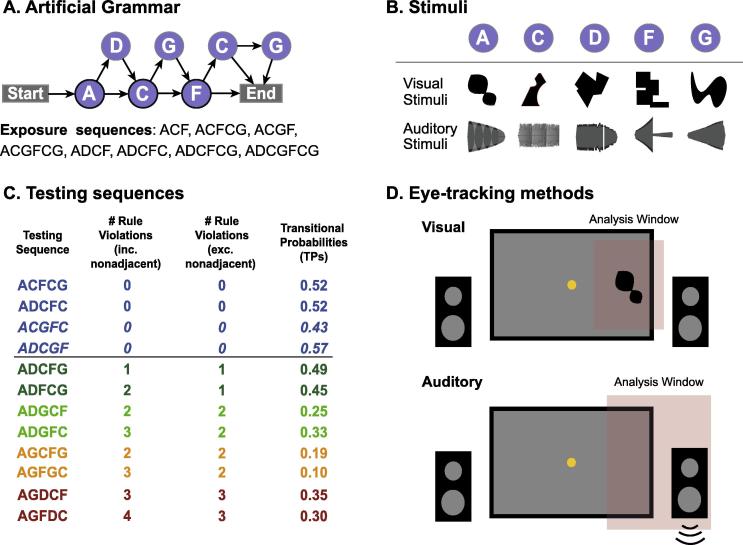
Artificial grammar learning paradigm. (A) Illustration of the Artificial Grammar (AG) used and the exposure sequences. Sequences are produced by following the arrows from the Start to the End. The AG contains five different elements which represent the shapes shown in (B, top) in the visual experiment and the computer generated sounds (B, bottom) in the auditory experiment. The sounds used are available in the supplementary materials. Eight exposure sequences were generated from the AG. These were presented during the exposure/refamiliarisation phases. During the subsequent testing phases, subjects were presented with the testing sequences (C) which were either ‘consistent’ with the AG (blue) or ‘violated’ the AG ordering relationships in specific ways (sequences below the black line). Two of the consistent sequences were familiar, having been heard in the exposure phase (top two sequences in blue). Two were novel consistent sequences (italicised in blue) that had not been presented during the exposure/refamiliarisation phases. The violation sequences contained different numbers of rule violations of both adjacent and nonadjacent relationships, as well as varying mean transitional probabilities (see Experimental procedures). The violation sequences were paired such that the two violation sequences in each pair (different coloured pairs of violation sequences) contained the same adjacent rule violations, but one of the sequences contained an additional nonadjacent rule violation. The duration of looking responses was recorded when the animals’ gaze fell within the analysis windows, illustrated in (D). In the auditory experiment, the analysis window includes all responses that exceed 3SDs of the variability in eye movements during the preceding baseline fixation period. In the visual experiment, the analysis window overlapped with the stimulus location. Responses were analysed when the gaze fell within ±10° vertical visual angle of the centre of the screen and within 12–32° of the centre of the screen in azimuth, where the visual stimulus was located. A, C and D adapted from (Wilson et al., 2013, Wilson et al., 2015b).

We used a mixed-complexity AGL paradigm (Fig. 1A) originally developed by Saffran and colleagues (2008), which we have previously used to evaluate auditory sequence processing using nonsense word stimuli in two species of monkeys and humans (Wilson et al., 2013, Wilson et al., 2015b). The AG was used to generate eight exposure sequences ranging from 3 to 7 elements in length (Fig. 1A). The sequence ordering relationships were identical in the auditory and visual experiments (see Fig. 1C, and below). The exposure sequences were used to expose the humans and monkeys to the sequence ordering relationships imposed by the AG.

After the exposure phase, a testing phase occurred during which we tested each participant’s behavioural sensitivity using the following set of testing sequences: The set of testing sequences contained four legal sequences, which were “consistent” with the artificial grammar. Two of these sequences had previously been presented during the exposure phase (familiar consistent) while the other two were novel consistent sequences, which had not been presented during the exposure phase (Fig. 1C). By comparing behavioural responses to these sets of consistent testing sequences we can evaluate whether familiarity or rote memorisation can explain the results (Wilson et al., 2013). The testing set of sequences also contained eight “violation” sequences that were inconsistent with the AG (Fig. 1C). All testing sequences were matched in length (consisting of five element sequences).

As previously reported (Wilson et al., 2015b), every legal, consistent sequence generated by this AG must follow a number of ‘rules’. Three rules govern the ordering relationships between certain adjacent elements in the sequences, as follows: ‘D’ must be preceded by ‘A’; ‘D’ must be followed by ‘C’; ‘G’ must be preceded by ‘C’. The artificial grammar also includes a nonadjacent rule, whereby ‘A’, ‘C’ and ‘F’ elements (Fig. 1C) must occur in that order but not necessarily with the elements occurring one after the other. The sequences violating the ordering relationships were designed to include increasing numbers of rule violations (Fig. 1C).

Another feature of the artificial grammar is that it contains a wide range of legal transitions between elements that occur with different frequencies and are therefore more or less predictable. The probability of one element being followed by another can be quantified by computing the transitional probabilities (TP), determined by the frequency with which a transition between adjacent elements occurs during the exposure phase relative to the frequency of that element occurring:

TP ofXtoY=P(Y|X)=frequency ofXY/frequency ofX

Illegal transitions are those that never occur during the exposure phase. These have a TP = 0 and, when present, reduce the average TP of the sequence. The mean TPs for each testing sequence are shown in Fig. 1C.

#### Auditory stimuli

Five computer generated sounds were created (www.bfxr.net, see Supplementary material). The corresponding waveforms of these sounds can be seen in Fig. 1B. The sounds were sampled at 22050 Hz, with sound amplitude onset and offset shaped by an 8 ms cosine ramp. The sound amplitudes were root-mean-square (RMS) balanced and fell well within the audible range of both species (Pfingst et al., 1978). Each sound was 410 ms in duration and sounds were combined into sequences with a 150 ms inter-stimulus interval using Matlab to produce testing sequences of 2650 ms duration.

#### Visual stimuli

Visual stimuli were abstract black shapes on a grey background (created in Adobe Photoshop), inspired by previous visual AG stimuli (Conway and Christiansen, 2006, Seitz et al., 2007, Osugi and Takeda, 2013). The height and width of the visual objects were the same for all shapes (9 cm × 9 cm, or approximately 8.5° visual angle). The shapes appeared serially in a sequence in the same location of the monitor, with the same timings as the auditory sequences (410 ms stimulus duration; 150 ms inter-stimulus interval).

### Macaque experiment

All macaque procedures performed were approved by the UK Home Office and comply with the Animal Scientific Procedures Act (1986) on the care and use of animals in research and also with the European Directive on the protection of animals used in research (2010/63/EU). We support the Animal Research Reporting of In Vivo Experiments (ARRIVE) principles on reporting animal research. All persons involved in this project were Home Office certified and the work was strictly regulated by the U.K. Home Office.

### Participants

Two male adult Rhesus macaques (*Macacca mulatta*), from a group-housed colony were tested (ages: M1 = 9 years, M2 = 4 years; weights: M1 = 12 kg, M2 = 7 kg). Both animals took part in both the auditory and visual experiments. Each animal had previously been trained on a visual fixation task and was acclimatized to head immobilisation. Head immobilisation was required so that high precision eye tracking data could be obtained throughout the testing sequences, which cannot yet be achieved with other approaches.

Given the ethical sensitivities involved in studying nonhuman primates and the 3Rs principles (one of which is on the *Reduction* of animal numbers), our work requires using the fewest macaques possible. A sample size of two is common in behavioural neuroscience experiments with macaques (e.g. Uhrig et al., 2014, Katz et al., 2016), provided that results are robust with each individual and that the effects generalize beyond one animal. Given that our results from several hundreds of trials with each animal are statistically robust and consistent between the two animals, there was little ethical justification to test additional monkeys. We discuss the implications of this ethical limitation in the discussion.

### Procedure

During each experiment the animal was seated in a primate chair in a sound-attenuating chamber (IAC Acoustics) 60 cm in front of a computer monitor. Stimuli were presented using Cortex Software (Salk Institute) and eye tracking data was recorded throughout the experiment (220 Hz infra-red eye tracker; Arrington Research). For further details regarding the eye tracking procedure see (Wilson et al., 2013). The animals were first tested on the auditory and then the visual experiment (see Discussion). In the auditory experiment, two audio speakers (Creative Gigawork T20, series II) were placed on either side of the monitor at ±46° visual angle. The sounds were presented at ∼75dB SPL (A weighting; calibrated with an XL2 sound level meter, NTI Audio). During the visual experiment, the high contrast visual monochromatic stimuli were presented sequentially on the screen subtending a visual angle of 8.5° horizontally and vertically. During the exposure phase, the visual stimuli were presented in the centre of the screen. During the test phase the sequences were presented on either the left or the right side of the screen, offset from the midline by ±15.2° (see Fig. 1D).

#### Exposure and refamiliarisation phase

The macaques were tested over several separate testing sessions: nine sessions (auditory experiment) and nine sessions (visual experiment) in Monkey 1 (M1); nine sessions (auditory experiment) and eight sessions (visual experiment) in M2. In each session, the monkeys participated in 1 to 6 testing runs, each of which was preceded by an exposure or refamiliarisation phase. Prior to the first testing run of the day, the animal was presented with the exposure sequences for 20 min (∼20 presentations of each exposure sequence in random order without resampling). In the auditory experiment, no responses were required during exposure or refamiliarisation. During exposure to the visual sequences, eye tracking was used to ensure that the animal was looking at the monitor, and they received a fluid reward after every exposure sequence to keep them motivated to look at the sequences. After the initial exposure phase, subsequent testing phases were preceded by a refamiliarisation phase. The sequences used for refamiliarisation were identical to those used during the exposure phase, but the length of exposure was shortened (eight repetitions of each exposure sequence, lasting approximately 8 min).

#### Testing phase

In both the visual and auditory experiments, each testing phase consisted of eight individual stimulus trials in which eight of the testing sequences were presented. To keep the individual testing runs within a session relatively brief and to ensure that an equal number of consistent and violation sequences were presented in each testing run, all four consistent sequences were presented with four of the eight possible violations sequences, in random order. In the subsequent testing run, the other four violation testing sequences were presented to ensure that all violation sequences were presented equally frequently with an equal number of consistent sequences.

Each trial began with the presentation of a fixation spot in the centre of the computer monitor. The monkey was required to fixate on the spot for 2 s, to centre their eyes. The initial central fixation acts as a baseline period used in the eye tracking data analysis. If the animal looked away from the fixation spot, the trial was aborted and restarted after a 2.5 s delay. If the monkey successfully fixated on the spot for 2 s, the fixation spot disappeared, the monkey was free to look around and the trial continued, as follows. To maintain the novelty of the stimulus presentations, and to encourage the monkeys to make looking responses to the stimuli, only 25% of successful fixation trials were followed by the presentation of a test sequence (‘stimulus trials’). On the other 75% of trials, no stimulus was presented. For all successful fixation trials, the monkey received a juice reward 5 s after the end of the fixation period, irrespective of whether or not a stimulus was presented. On stimulus trials, in the auditory experiment a pseudo-randomly selected sequence was presented from either the left or the right audio speaker. In the visual experiment, a stimulus sequence was presented on the left or right side of the monitor. In both experiments, the animal was free to look around during this part of the trial, and the animal’s eye position was recorded for 5 s (for a total of 7 s of eye tracking data, including the 2 s baseline fixation period and 2.65 s stimulus presentation period). The next trial began after a 4 s inter-trial interval. After each testing run containing eight testing sequences, the animal was refamiliarised with the exposure sequences before the next testing run began. The total numbers of successfully completed fixation trials followed by a stimulation trial that were available for analysis were 464 (M1 = 240, M2 = 224) for the visual experiment and 480 (M1 = 232, M2 = 248) for the auditory experiment.

### Data analysis

**Fig. 2:**
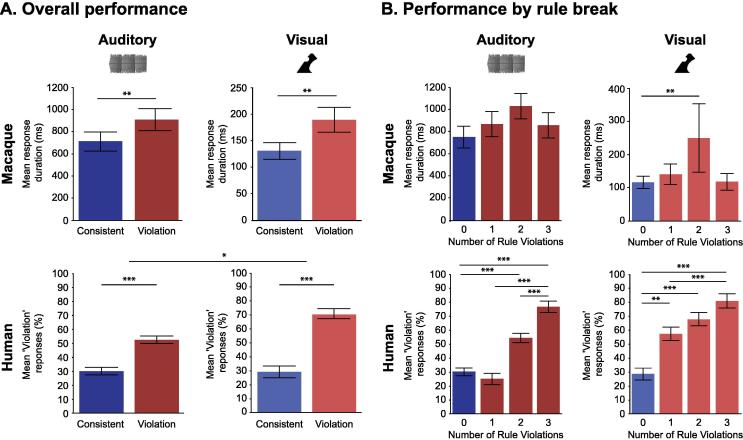
Macaque and human performance as a function of sequence type. (A) Macaque (top) and human (bottom) behavioural responses to auditory and visual consistent and violation sequences (left and right of each panel respectively). (A, top panel; macaques) Mean (±standard error of the mean, SEM) looking response durations in response to sequences that were either consistent with the Artificial Grammar (AG, in blue) or violated the AG ordering relationships (red). (A, bottom panel; humans) Mean (±SEM) proportion of trials on which participants classified consistent and violation sequences as containing “violations”. (B) Mean (±SEM) looking response durations (in macaques, top panel) and proportion of “violation” responses (in humans, bottom panel) subdivided by the number of adjacent rule violations that the sequences contained. Significance levels represented by ^***^p < 0.001, ^**^p < 0.01, ^*^p < 0.05.

The eye tracking data for each trial contained both the 2 s baseline fixation period during which the animal fixated on the central fixation spot and the subsequent 5 s stimulus period during which the test sequence was presented (Fig. 2B). In the auditory experiment, to calculate the duration of looking responses towards the presenting audio speaker, we initially calculated the baseline variability in the eye movement during the final 1.5 s of the fixation period, immediately preceding stimulus presentation. The initial 0.5 s period after the onset of the fixation spot was excluded from analysis because during this period the monkey saccades to the fixation spot. Looking responses to the test sequences were defined individually for each animal as looks toward the presenting audio speaker (left or right) exceeding 3 SD of the variability in the baseline fixation period (for further details see Wilson et al., 2013). In the visual experiment, responses to the visual sequences were recorded when the gaze fell within an analytical inclusion window, defined within the range of ±10° elevation and 12–35° azimuth, centred around the visual stimuli location (see Fig. 1D).

Analyses comparing consistent and violation sequences, and those assessing nonadjacent rule violations, were conducted using a repeated measures (RM) ANOVA. For analyses based on the number of rule violations a univariate ANOVA was used because only four of the violation sequences appeared in each test run, precluding a repeated measures analysis for the number of rule violations factor. For the univariate ANOVA normality assumptions were met. For the RM-ANOVA normality assumptions were not always met (e.g., one of the animals had less normally distributed results), but because there is no suitable non-parametric alternative we opted to use the RM-ANOVA to account for the within subject variability in the condition and nonadjacent rule violation factors. A partial correlation (controlling for animal specific variability) was also used to compare look durations against the average transitional probabilities of the sequences in each modality. To assess whether mean transitional probabilities were more strongly correlated to looking response durations in one modality than the other we used a Fisher *r*-to-*z* transformation. This transformation takes into account the correlation *r* value and the sample size to compute a *z* value to evaluate significant differences between the correlations in each modality.

In the human experiments (see below) RM-ANOVAs were used to directly compare results in the visual and auditory modality. It was not possible to make this comparison in the monkey results since the eye tracking data had to be collected in different ways: responses beyond a certain threshold in the auditory experiment but within a predetermined field of view in the visual experiment. Nonetheless, the pattern of behavioural responses given to the auditory and visual stimuli was very similar between modalities, and a direct test across the sensory modalities to assess the association between sequence transitional probabilities and behavioural performance showed no significant modality difference (see Results section on monkey transitional probabilities).

### Human experiment

Human participants provided informed consent to participate in this study, which was approved by the human studies Ethical Review Body at Newcastle University and conformed with the 2013 WMA Declaration of Helsinki. Participants either received course credits or £5 to compensate them for their time.

### Participants

Human participants (19; 11 female, 8 male; mean age 24 years) were tested on the visual experiment. Another cohort of participants (19; 13 female, 6 male; mean age 31 years) was tested on the auditory experiment. Participants were recruited separately for each experiment via the Newcastle University Institute of Neuroscience participant pool. All participants had normal or corrected-to-normal vision and hearing, and none reported any language or attention disorders. One participant in the visual experiment had to be excluded from the group results (not included in the above numbers) because they self-reported having previously suffered from epilepsy, which although having been surgically treated raises questions about how normative their performance may be.

### Procedure

Participants were seated 60 cm in front of a computer monitor in a psychophysics laboratory. During the auditory experiment, stimuli were presented through Sennheiser HD202 headphones at ∼75 dB SPL (sound calibration procedure as above for the monkey auditory experiment). For the visual experiment, the shapes appeared serially in the centre of the grey screen subtending a visual angle of approximately 8.5°. The experiment was controlled using Matlab scripts running the Psychophysics Toolbox: http://psychtoolbox.org.

#### Exposure and refamiliarisation phase

Participants were initially exposed to sequences that followed the AG sequence ordering relationships (Fig. 1B). They were asked to listen to or watch the exposure sequences. Each of the 8 exposure sequences was presented in random order without resampling. Each exposure sequence was repeated six times (48 sequences in total, total exposure period duration of 5 min). After the initial testing run, each subsequent testing phase was preceded by a refamiliarisation phase in which the same exposure sequences were present in random order, with each sequence repeated four times (32 presentations).

#### Testing phase

Each exposure phase was followed by a testing phase in which 32 testing sequences were presented. Each of the eight violation testing sequences were presented twice during the run, and each of the four consistent sequences was repeated four times, to ensure that the numbers of consistent and violation sequences were matched in each testing run. The testing sequences were presented without resampling in a random order. Participants were instructed to respond after the testing sequence had been completely presented by pressing one of two keys on the keyboard to indicate that the sequence they had just heard ‘followed the same pattern’ as the sequences heard during the exposure phase (consistent) or ‘did not follow the pattern’ (violation). All testing sequences were 5 elements long, and the human participants were informed at the start of the experiment that the sequence length was not a relevant cue. A 2 s inter-trial interval followed the participant’s response before the next testing trial began. After each testing run there was a brief break, followed by a refamiliarisation phase and another testing run until a total of 4 testing runs had been completed. Each participant completed 128 trials. The decision to use a forced choice task with the humans rather than eye tracking was made following previous experiments by the laboratory, which demonstrated that sequence learning effects are difficult to assess with eye tracking in adult humans but can be assessed with an explicit task such as the one used here (see Wilson et al., 2015b; also see discussion).

### Stimulus labelling

Although the auditory and visual stimuli were designed to be sounds or images that are difficult to label, with the human participants we empirically assessed whether a stimulus labelling strategy could have assisted their performance. If so, this could contribute to potential cross-species differences in performance given that it is not possible to know whether the monkeys used a similar strategy. After the experiment, the human participants were asked in a debriefing questionnaire whether they relied on verbally labelling either the visual or auditory stimuli. Although many of the participants stated that they relied on sound or picture labelling (Visual: 10/19; Auditory 9/18; one participant did not complete the debriefing questionnaire), using a labelling strategy did not appear to significantly aid performance (independent samples *t*-test, performance of labeller vs non-labeller: Visual: *t_17_* = 0.465, *p* = 0.648; Auditory: *t_16_* = 1.90, *p* = 0.075).

### Data analysis

In these forced-choice experiments, the human participants gave binary responses that a sequence “follows the pattern” or “does not follow the pattern”. By comparison, the monkey data are based on looking durations, and we analysed whether the animals look longer to the violation than consistent sequences. To facilitate analysis of the human data as comparably as possible across the species, we calculated the percentage of “violation” responses given to the violation or consistent sequences (trials in which they indicated that the sequence “does not follow the pattern”).

Using the percentage of violation responses, paired samples *t*-tests were used to confirm that participants were able to discriminate between the consistent and violation sequences in each modality. RM-ANOVAs were used to compare the main effects across modalities, to assess the factor for number of rule violations (*0, 1, 2, 3*) and to explore the effect of the nonadjacent rule violation. All post-hoc tests were Bonferroni corrected. Pearson correlations were used to compare performance as a function of sequence transitional probabilities. Normality assumptions for each of these tests were met by the data.

## Results

### Macaque results

In separate experiments, macaque monkeys were tested with sequences of auditory or visual stimuli. Initially, the macaques were exposed to a subset of the sequences generated by the artificial grammar (AG). In a subsequent testing phase the monkeys were tested with sequences which were either consistent with the AG or contained specific violations of the AG ordering relationships. Test sequences were presented from the animals’ left or right part of space, either from the left or right audio speaker or on the left or right sides of the monitor, respectively in the auditory and visual experiments. Eye tracking data was recorded, and the duration of looking responses towards the stimuli were objectively and automatically calculated. Look durations for each condition were averaged over testing runs and mean look durations were entered into the analyses below. When the data from individual trials were analysed using linear mixed models, highly consistent results were obtained.

#### Main effects

To explore whether the macaques produced longer looking responses to sequences containing ordering violations, for each modality a repeated measures (RM) ANOVA was conducted with a within-subjects factor of condition (*consistent* vs *violation*) and a between-subjects factor of macaque (*M1* vs *M2*). The dependent variable was the mean looking duration for each run. In both the visual and the auditory experiments the macaques spent longer looking towards the violation sequences than the consistent sequences (Main effect of condition: Auditory: *F*_1,58_ = 12.41; *p* = 0.001; Visual: *F*_1,56_ = 11.81; *p* = 0.001, Fig. 2A). This finding was consistent in both animals, shown by a lack of an interaction between the factors of condition and macaque (Auditory: *F*_1,56_ = 0.29; *p* = 0.594; Visual: *F*_1,56_ = 0.32; *p* = 0.573). To ensure that the effect could not be attributed solely to sequence familiarity (i.e., the animals responding more strongly to unfamiliar sequences that they had not been exposed to), we separated the consistent testing sequences used into those which were ‘familiar’ (heard during the exposure phase) and those which were ‘novel’ (not heard during exposure). We analysed responses to these subsets of sequences using an RM-ANOVA with a within-subjects factor of condition with three levels (*novel consistent*, *familiar consistent* or *violation sequence* type) and a between-subjects factor of macaque (*M1* vs *M2*). This confirmed the observed main effects of condition in both sensory modalities (Auditory: *F*_2,116_ = 6.53, *p* = 0.002; Visual: *F*_2,112_ = 6.47, *p* = 0.002) with no significant interaction of condition and macaque factors (Auditory: *F*_2,116_ = 0.49, *p* = 0.614; Visual: *F*_2,112_ = 0.240, *p* = 0.787). Bonferroni corrected post-hoc tests confirmed that there were no differences in how the animals responded to the novel and familiar consistent sequences (*p* = 1 in both cases), and that the animals responded to both of these for significantly shorter durations than the violation sequences in both modalities (*p* < 0.05 in all cases). These results demonstrate that macaques respond to ordering violations in both visual and auditory sequences. Moreover, these responses cannot be attributed simply to attenuated responses to the familiar sequences that the animals heard or saw repeatedly during the exposure phase, in either modality: The behavioural results indicate that the auditory and visual sequences were treated comparably to novel (unfamiliar) consistent sequences, but differently to the violation sequences. Finally, we assessed whether learning effects might become more pronounced with repeated testing. However, over repeated testing runs, the difference in look response durations to violation vs consistent sequences either remained constant or decreased in both animals (auditory modality: M1: *r* = 0.49, *p* = 0.397, M2: *r* = −0.372, *p* = 0.023; visual modality: M1: *r* = −0.474, *p* = 0.004; M2: *r* = 0.142, *p* = 0.236). These results suggest that sensitivity to the violation sequences did not take multiple runs to develop and did not result in an increase in performance over time or across sensory modalities. To further assess what properties of the sequences the monkeys are sensitive to across the two modalities, we conducted several further analyses.

#### Number of rule violations

Next, we investigated the responses to sequences containing different numbers of rule violations and how the patterns of responses varied across modalities. The test sequences were categorised according to the number of adjacent rule violations that they contained (0, 1, 2 or 3 violations, excluding nonadjacent rule violations, Fig. 1C; see Experimental procedures). While one might predict continuously increasing looking responses to sequences containing higher numbers of rule violations, Fig. 2B shows that in both modalities the monkeys responded maximally to sequences containing two violations. This pattern is remarkably similarly across the two sensory modalities. In both modalities a univariate ANOVA was conducted with a dependent variable of looking duration and the factors ‘number of rule violations’ (*0*, *1*, *2* or *3*) and macaque (*M1* or *M2*). For the visual experiment there was a main effect of number of rule violations (*F*_3,174_ = 3.86, *p* = 0.010, Fig. 2B), demonstrating that the monkeys’ responses varied based on the number of rule violations within a testing sequence. In the auditory experiment, a weaker but similar effect is seen (Fig. 2B) with a statistical trend for the number of rule violations (*F*_3,179_ = 2.16, *p* = 0.094). As with the visual experiment results, the longest looking durations were elicited by sequences containing two rule violations. Post-hoc comparisons supported these observations where Bonferroni corrected tests revealed significantly longer responses to sequences with two violations than sequences containing zero violations in the visual modality (*p* = 0.015) and a statistical trend in the auditory modality (*p* = 0.087). In the visual modality, responses to sequences with two violations were also significantly longer than the sequences containing three violations (*p* = 0.037). There was no interaction between number of rule violations and monkey in either modality (in both cases, *p* > 0.1). Thus, the pattern of results is largely similar across the modalities, showing the greatest looking responses to sequences that contain two violations. Moreover, the auditory results closely mirror the effects previously reported using the same artificial grammar, but in that case sequences of nonsense words were used, which serves as a point of reference for the auditory effects reported here (Wilson et al., 2015b).

#### Transitional probabilities

**Fig. 3:**
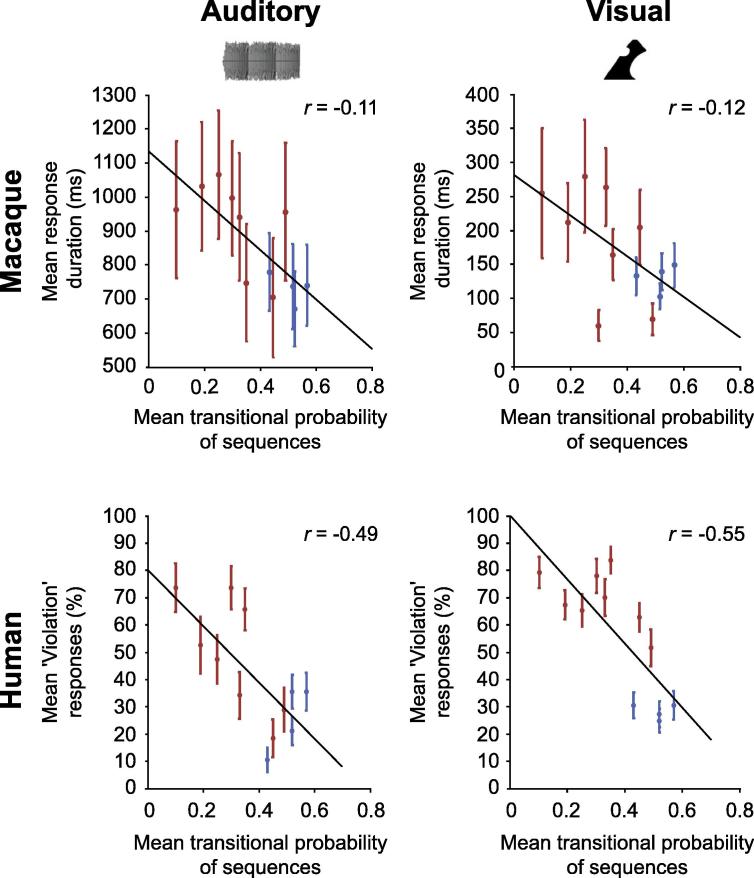
Macaque and human behaviour as a function of statistical regularities (transitional probabilities). (Top panel; macaques) Mean (±SEM) looking response durations, plotted against the mean transitional probability of each consistent (blue) and violation (red) sequence (see Fig. 1C). (Bottom panel; humans) mean percentage of violation responses plotted in the same format as for macaques.

To explore how the monkeys responded to the statistical regularities in the sequences established during the exposure phase, responses were analysed relative to the mean transitional probabilities of the testing sequences (Fig. 1B). Partial correlations, controlling for animal specific variability, showed a significant negative correlation between look duration and average TP in both modalities (Auditory: *r* *=* *−*0.112, *p* = 0.014; Visual: *r* *=* *−*0.120, *p* = 0.008, Fig. 3). These results support previous findings in the auditory modality (Wilson et al., 2015b), demonstrating that macaques look longer to sequences with lower average transitional probabilities (i.e., those containing greater numbers of unexpected transitions). This observation demonstrates that the predictable regularities established via statistical learning during the exposure phase are a strong explanatory factor in the sequence processing behaviour of nonhuman primates. Furthermore, to compare whether the correlation between response duration and mean transitional probability might be stronger in the visual or auditory experiments, we compared the correlation coefficients for the two modalities using a Fisher *r*-to-*z* transformation. This analysis revealed no significant difference in the magnitude of the correlation in the auditory and visual modality (*z* *=* 0.12, *p* = 0.45). These effects related to the statistical regularities in the sequences provide further support of a comparable pattern of macaque behavioural responses in the two sensory modalities.

#### Nonadjacent rule violations

**Fig. 4:**
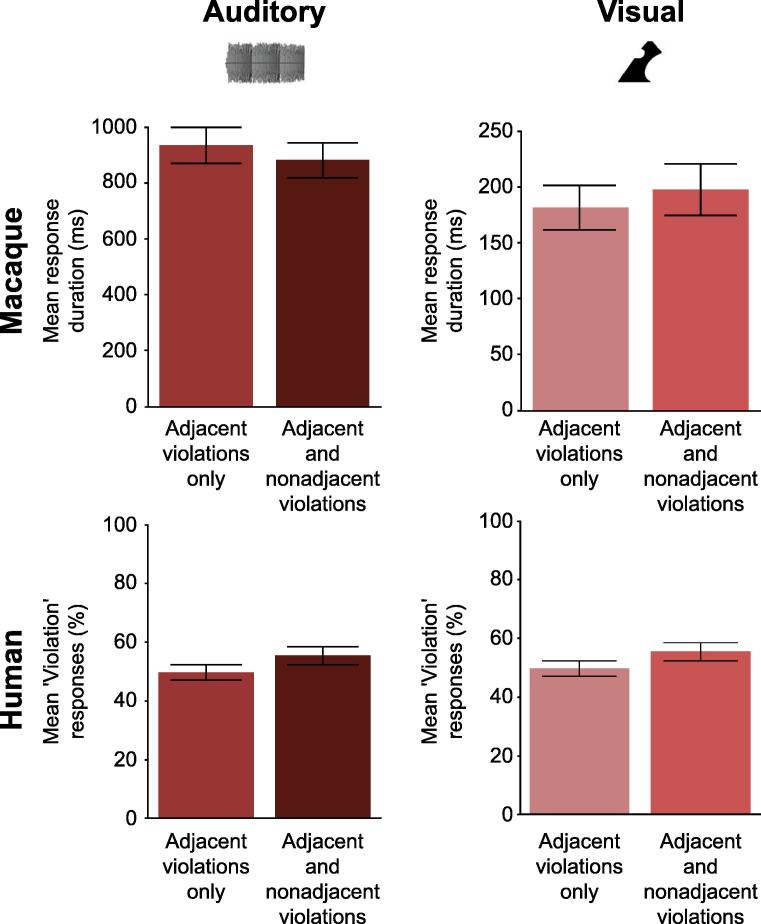
Macaque and human performance: nonadjacent rule violation. (Top panel; macaques) Mean (±SEM) looking response durations to violation sequences that only contained local violations but not violations of the nonadjacent relationship (red, see Experimental procedures), contrasted to sequences that are matched in the local violations but that also violate the nonadjacent association (dark red) between the ‘A’, ‘C’ or ‘F’ elements in the AG (Fig. 1A). (Bottom panel; humans) Mean (±SEM) percentage of violation responses to the identical testing sequences.

Finally, we investigated whether the presence of a nonadjacent sequence order violation produces longer looking responses in either the visual or auditory modality. An RM-ANOVA was conducted with factors of nonadjacent rule (nonadjacent rule *intact* vs *broken*; with both sets of sequences matched in the number of local transitions), sequence pair (*1*, *2*, *3*, *4*) and macaque (*M1* vs *M2*) in both the auditory and visual modalities. There was no significant sensitivity to the nonadjacent rule violation in either modality (main effect of nonadjacent rule: Visual: *F*_1,216_ = 0.399, *p* = 0.528; Auditory: *F*_1,216_ = 0.518, *p* = 0.472, Fig. 4) and no interaction with macaque (*p* > 0.05). These results replicate those previously reported in macaques in the auditory modality for the nonadjacent sequencing relationship (Wilson et al., 2013, Wilson et al., 2015b), suggesting that in this artificial grammar with multiple cues to ‘grammaticality’, the nonadjacent relationship may not be noticed by macaques.

### Human results

Human participants were tested with sequences of auditory and visual stimuli identical to those used to test the monkeys. Following exposure to sequences generated by the AG, human participants were tested with a two-alternative forced-choice task. Participants were presented with a randomly selected testing sequence and asked to judge if the sequence “followed the same pattern” as those in the exposure period or “violated the pattern”. To ensure that analyses were comparable to those conducted for the monkey data (where we measured the duration of looking responses elicited by the testing sequences), we analysed the proportion of “violation” responses that the human participants made to the testing sequences. This allowed a more direct cross-species comparison of responses than defining performance relative to chance levels in the human data, which is not possible in the same way in the monkey data (see Experimental procedures).

#### Main effects

The human participants were sensitive to the sequencing relationships, correctly identifying consistent and violation sequences in both modalities (paired sample *t*-tests, auditory: *t*_18_ = 5.84, *p* < 0.001; visual: *t*_18_ = 5.77, *p* < 0.001, Fig. 2A). An RM-ANOVA with a within-subjects factor of sequence condition (*violation* vs *consistent*) and a between- subjects factor of modality supported these results (main effect of condition, *F_1,36_* = 62.25, *p* < 0.001). This analysis revealed somewhat better performance in the visual relative to the auditory experiment (interaction with modality *F_1,36_* = 4.69, *p* = 0.037, Fig. 2A).

#### Number of rule violations

An RM-ANOVA with a dependent variable “proportion of violation responses” and the within-subjects factor of number of rule violations (*0*, *1*, *2* or *3*) was conducted for the auditory and visual experiment. In both experiments there was a main effect of number of rule violations (auditory: *F_3,54_* = 48.31, *p* < 0.001; visual: *F_3,54_* = 22.30, *p* < 0.001), with more violation responses given to sequences with higher numbers of rule violations. This pattern of responses was highly comparable across the two sensory modalities (Fig. 2B). Moreover, the human auditory pattern of results is consistent with a previous study using the same artificial grammar and nonsense word sounds (compare Fig. 2B, and Wilson et al., 2015b). Bonferroni corrected post hoc tests were conducted to identify differences in responses between sequences containing different numbers of rule violations. In the visual experiment, differences were observed between all sets of sequences (*p* < 0.05), except 1 vs 2 violations (*p* = 0.116), and 2 vs 3 violations (*p* = 0.086). In the auditory experiment, differences were observed between all sequence types (*p* < 0.001), except between sequences with 0 vs 1 violation (*p* = 1.0). These results show largely similar patterns of results between the auditory and visual experiments, with quantitative differences in effects between sequences with comparable numbers of violations (see Fig. 2B). For instance, the lack of any difference between sequences containing zero and one rule violation in the auditory experiment might suggest participants are less sensitive to subtle rule violations in the auditory than the visual modality.

#### Transitional probabilities

In both modalities, human participants were significantly better at detecting violations of sequences with more unexpected transitions and lower transitional probabilities (Pearson’s correlation: auditory experiment: *r* = −0.47, *p* < 0.001; visual experiment: *r* = −0.552, *p* < 0.001, Fig. 3). There was no difference in the magnitude of this correlation between the modalities (Fisher *r*-to-*z* transformation: *z* = −0.51, *p* = 0.305).

#### Nonadjacent rule violations

There was no evidence that human participants detected the nonadjacent rule violation. An RM−ANOVA with a within-subjects factor of nonadjacent rule (nonadjacent rule *intact* vs. nonadjacent rule *broken*) and of sequence pair (*1*, *2*, *3*, *4*) showed no main effect of the nonadjacent rule in either modality (auditory: *F_1,18_* = 0.073, *p* = 0.790; visual: *F_1,18_* = 1.564, *p* = 0.227, Fig. 4). This result suggests that, as with the monkeys, when presented with a mixed complexity AG containing many adjacent sequencing relationships, monkeys and humans tend to miss the nonadjacent relationship, even though it is present in every consistent sequence.

## Discussion

This study aimed to shed light on whether the system supporting human auditory and visual sequence processing, which has been linked to certain language-related operations, evolved out of a similarly multisensory system shared by nonhuman primates, or whether the multisensory aspects of this system are a more recent evolutionary specialisation in humans. We investigated this using identically structured sequences of auditory and visual AG sequences in humans and macaque monkeys. Both species were sensitive to violations of the sequence ordering relationships regardless of whether the sequence elements were abstract shapes or computer-generated sounds. The auditory results recapitulate previous findings in the auditory modality using sequences of nonsense words generated by the same artificial grammar (Wilson et al., 2013, Wilson et al., 2015b). Critically, this study went beyond previous experiments by directly comparing human and monkey responses to identically constructed sequences consisting of auditory or visual stimuli. Overall, the pattern of results, including those relating to the sequence transitional probabilities, rule violations and sensitivity to a nonadjacent rule, provide considerable evidence for similar patterns of responses across the sensory modalities in humans and monkeys, with a few quantitative differences within and across the species, as we consider.

### Cross-species and cross-modality similarities

Humans and monkeys showed stronger responses to sequences containing less predictable transitions, defined as the average pairwise transitional probabilities (TPs) of the sequences (Fig. 3). TPs reflect the likelihood that one element will transition to another, calculated from the frequency of pairwise transitions experienced during the exposure phase. These observations are consistent with previous work on statistical learning in human and nonhuman animals (Saffran et al., 1996, Kirkham et al., 2002, Wilson et al., 2013, Wilson et al., 2015b), which together support the notion that sensitivity to statistical regularities is an important feature of the sequence ordering capacities of humans and other animals. Importantly, here we show that this pattern of increased responses to less predictable sequences is highly comparable between the auditory and visual modalities in both humans and macaque monkeys (compare human and macaque behaviour in Fig. 3).

Furthermore, the testing sequences in this experiment were classified into subtypes, based on how many adjacent rule violations they contained (see Wilson et al., 2015b methods, and Fig. 1). Although there were some interesting subtle cross-species differences, which we consider in more detail in the next section, within each species the general pattern of responses to the auditory and visual sequences was strikingly similar (compare auditory and visual performance in Fig. 2). This provides evidence of broadly comparable processing across modalities, in both humans and monkeys.

Finally, neither species appeared to detect violations of the nonadjacent sequencing relationship, in either the auditory or visual modality. In a previous study using auditory nonsense word stimuli, a minority of human participants did show sensitivity to this nonadjacent relationship, however this sensitivity was not observed in macaques or many of the human participants (Wilson et al., 2015b). This does not imply that monkeys are unable to learn nonadjacent relationships. In the absence of informative adjacent sequence relationships, previous studies have identified sensitivity to nonadjacent dependencies in nonhuman primates (e.g. see Newport and Aslin, 2004, Newport et al., 2004, Ravignani et al., 2013, Milne et al., 2016). Importantly, the finding that neither species showed sensitivity to the nonadjacent violations in the current auditory or visual experiments again underscores the overall similarity of effects across the two sensory modalities.

### Differences between species and modalities

The analyses of responses to sequences containing increasing numbers of rule violations, although similar across the sensory modalities within each of the species, showed some interesting cross-species differences, as follows. The rules used in these analyses were defined as the relationships that must be followed by every legal sequence, for example, if ‘D’ is present, it must always be followed by ‘C’ (Wilson et al., 2015b). These relationships occur consistently and were therefore hypothesised to be more salient than the less predictable, more variable relationships (e.g., ‘C’ can be followed by ‘G’, ‘F’ or the end of the sequence). The human results showed a linear increase in sensitivity with the number of rule violations, whereas in the monkeys, the strongest looking responses were given to sequences with two rather than three rule violations in both the visual and auditory modalities (Fig. 2). These results are identical to those previously reported using the same AG with nonsense word stimuli in both humans and monkeys (Wilson et al., 2015b). This represents an intriguing cross-species difference that was not evident in the other behavioural results, such as those based on measures of transitional probabilities. Two of the rules involve assessing backwards relationships about which elements can legally precede others (i.e. ‘D’ must be preceded by ‘A’). The average transitional probabilities of the sequences, calculated forward from the beginning to the end of the sequences, are by definition not sensitive to these rules. It therefore appears that the monkeys may be less sensitive to these backwards relationships, which appear to be salient to the humans.

The human participants showed broadly comparable patterns of responses in the auditory and visual modality. However, there were some notable quantitative differences in their behaviour. In the visual experiment, participants were sensitive to sequences containing even a single rule violation, while in the auditory modality only sequences containing at least two violations could be discriminated from consistent sequences (Fig. 2). These differences may stem from auditory stimulia being dynamic and possibly more fleeting, whereas our visual stimuli consisted of a sequence of static images, potentially leading to the observed cross-modal differences in the capacity to encode and hold the sensory information in memory (Fritz et al., 2005, Cohen et al., 2009, Schulze et al., 2012).

### Methodological differences in testing humans and monkeys

Unavoidably, the behaviour of both species was measured in different ways. We have previously attempted to test humans and monkeys using identical eye tracking approaches. However, this approach, which can be used in infants and nonhuman animals to measure sequence learning effects, was not sensitive enough to measure effects in adult humans, who otherwise show effects when an explicit task is used (see Wilson et al. (2015b), Supp. Info.). Implicit artificial grammar learning tasks have been carried out in adult humans, for example, using cover tasks (Turk-Browne et al., 2005) or rapid serial visual presentations (Kim et al., 2009), but these tasks are difficult to conduct in nonhuman primates because they require considerable operant conditioning and training (Heimbauer et al., 2012).

However, although the subtle behavioural differences observed in our results might be explained by any of the methodological differences in testing between the species, the similarities in the results observed cannot be explained so easily and are thus all the more remarkable. Instead the highly similar pattern of results observed in the auditory and visual experiments suggest both species appear to rely on comparable mechanisms for processing sequences across the sensory modalities.

We tested both monkeys on the auditory task first. To assess whether this contributed to carry-over effects from the auditory to the visual task, we conducted additional analyses of the monkeys’ responses over the multiple testing runs. If learning effects persisted across testing runs, we might predict stronger responses to violation than consistent sequences in later runs, when there has been more opportunity to learn the sequencing relationships. However, performance did not increase over time, instead either remaining constant or decreasing, suggesting that learning effects did not accumulate over multiple testing runs. Moreover, if learning did persist and carry over to the second (visual) experiment, this might predict a boost in performance in the initial testing runs on the visual experiment relative to the auditory task. However, we see little evidence for such carry-over effects. These observations are consistent with findings in humans that cross-sensory sequence learning transfer effects are surprisingly limited for various types of sequencing relationships (Conway and Christiansen, 2005, Conway and Christiansen, 2006, Mitchel and Weiss, 2011, Frost et al., 2015, Walk and Conway, 2016).

In the human experiment it was possible to test several participants. However, due to ethical constraints it was only possible to test two macaques, which is a common approach in behavioural and neuroscience studies with nonhuman primates (see Experimental procedures). Therefore, the monkey results should be considered as a two-subject case study. Nonetheless, despite this small sample size a large amount of data was collected (several hundred testing trials per macaque) and these produced statistically robust results that were strikingly similar in both animals. Moreover, despite the sample size differences, very similar patterns of responses were observed in both species. Therefore, our results suggest that these multi-sensory sequence processing abilities are not unique to humans or to language.

### What aspects of the sequencing relationships are humans and monkeys learning?

It is important to consider what the behavioural results tell us about the types of sequencing relationships that are learned and how this might vary across species or sensory modalities. Our experimental paradigm was designed to assess whether either species simply memorised the sequences presented during the exposure period, which was done by testing with both ‘familiar’ consistent sequences that were presented during the exposure phase and ‘novel’ consistent sequences. Neither macaques nor humans responded differently to these novel sequences in either modality (see also Wilson et al., 2013, Wilson et al., 2015b), suggesting that learning cannot be attributed to familiarity or rote memorisation of entire sequences. Thus, neither species appears to encode or process the sequences at the level of whole strings, by matching entire sequences to those heard during exposure to assess similarity (Beckers et al., 2016). Moreover, we see no evidence that either the monkeys or the human participants learned long distance relationships between non-adjacent sequence elements (Fig. 4). Rather, these results, and those of previous experiments using similar stimuli (Wilson et al., 2015b), suggest that the most parsimonious explanation is an account based on monitoring the pairwise relationships between adjacent sequence elements during the exposure phase. This interpretation is compatible with a statistical learning account, that participants respond based on the probabilities with which each element in a sequence predicts the next (e.g., transitional probabilities, Fig. 3), or that humans and monkeys respond to the frequency with which adjacent pairs of elements (bigrams) co-occurred during exposure. The results support the notion that both humans and monkeys respond in highly comparable ways to the adjacent sequencing relationships in the auditory and visual sequences.

### Comparable mechanisms for auditory and visual sequence learning in primates

The evidence from this study points to sequence processing engaging comparable mechanisms across the sensory modalities in monkeys and humans. The observed cross-species differences in sensitivity to rule violations, although providing additional insights into the bases for the behavioural responses in the two species, do not challenge this interpretation because of the remarkable similarity of the responses across the sensory modalities in both humans and monkeys.

The neural basis for sensory sequence processing across modalities in nonhuman animals remains unclear (Frost et al., 2015). Recent neuroimaging and neurophysiological studies have identified brain regions and neural responses that show correspondences in effects during various forms of sequence processing between humans and monkeys (Wang et al., 2015, Wilson et al., 2015a, Milne et al., 2016, Kikuchi et al., 2017), including frontal cortex areas known to be involved in certain syntactic operations in humans (Friederici, 2011, Nelson et al., 2017). Many human neuroimaging studies have used spoken or written language as stimuli and report overlapping regions, including frontal cortex, which are involved in language-processing (Friederici, 2011, Bemis and Pylkkänen, 2012). In nonhuman animals, data from neuronal recording studies during sequence processing are also available in the auditory (Lu and Vicario, 2014, Kikuchi et al., 2017) and visual modalities (Meyer and Olson, 2011). Furthermore, a variety of human neuroimaging experiments and neuronal recording studies in animal models show multisensory interactions between individual auditory and visual objects, which supports the notion of brain interactions being highly multisensory (Jordan et al., 2005, Budinger et al., 2006, Ghazanfar and Schroeder, 2006, Stein and Stanford, 2008, Chandrasekaran et al., 2009, Leavens et al., 2010, Cohen et al., 2011, Romanski, 2012, Soto-Faraco et al., 2012). However, direct behavioural comparisons of humans and nonhuman primate sequence processing abilities across sensory modalities were missing. It thus remained uncertain whether similar operations occur across the sensory modalities and how comparable they are across the species.

Our behavioural results can be used to constrain predictions about the possible neural mechanisms for auditory and visual sequence processing in nonhuman primates. The findings suggest that sequence processing in both modalities is served by similar neurobiological operations, although future neurobiological study will be required to identify the streams of processing involved in auditory and visual sequence processing, and how they interact. Endeavours to obtain comparative behavioural and neurobiological data on cross-sensory sequence processing can provide important insights into the evolutionary origins of human communication as a multisensory system (Fitch, 2010). The evolutionarily conserved aspects could be modelled at the neuronal level in nonhuman animals (e.g. Vicario and Yohay, 1993, Meyer and Olson, 2011, Kikuchi et al., 2017).

## Conclusions

Our results demonstrate that both humans and monkeys are comparably sensitive to the ordering relationships between adjacent elements in sequences of auditory and visual stimuli. Moreover, in both species the patterns of responses are highly similar across the sensory modalities, suggesting that sequence processing might be supported by similar neural mechanisms in different sensory domains. The comparative findings point to sequencing operations being evolutionarily conserved in human and nonhuman primates, and are therefore unlikely to have been a recent adaptation for language in humans. The findings from this behavioural study raise intriguing questions about the neural substrates supporting these abilities, and they pave the way for the corresponding neuronal processes to be studied in macaques as a neurobiological model system.

## Author contributions

Conceived and designed the experiments: AEM, CIP and BW. Performed the experiments: AEM. Analysed the data: AEM. Wrote the paper: AEM, CIP and BW.

## Appendix A Supplementary data

Supplementary audio 1

Supplementary audio 2

Supplementary audio 3

Supplementary audio 4

Supplementary audio 5
